# Mineral, seed morphology, and agronomic characteristics of proso millet grown in the inland Pacific Northwest

**DOI:** 10.3389/fnut.2024.1394136

**Published:** 2024-09-11

**Authors:** Tayler Reinman, Jessica Braden, Nathan Daniel Miller, Kevin M. Murphy

**Affiliations:** ^1^Sustainable Seed Systems Lab, Department of Crop and Soil Science, Washington States University, Pullman, WA, United States; ^2^Spalding Lab, Department of Botany, University of Wisconsin, Madison, WI, United States

**Keywords:** nutritional characteristics, end-use characteristics, underutilized crops, crop diversity, variety trials, climate resilience

## Abstract

Climate change increases stressors that will challenge the resiliency of global agricultural production. Just three crops, wheat, maize, and rice, are estimated to sustain 50% of the caloric demand of the world population, meaning that significant loss of any of these crops would threaten global food security. However, increasing cropping system diversity can create a more resilient food system. One crop that could add diversity to wheat-dominated cropping systems in the inland Pacific Northwest is proso millet, a climate-resilient, small-seeded cereal crop that is highly water efficient, able to grow in low fertility soils, and has a desirable nutritional profile. Proso millet shows potential for adoption in this region due to its short growing season, compatibility with regional equipment, and environmental requirements, however US cultivars have been developed for the Great Plains and little research has been conducted outside of this region. To better understand the potential for adoption in the inland PNW, seven commercially available varieties were planted in a researcher-run trial in Pullman, WA and in a series of producer-run trials across the region in 2022. Samples were analyzed for mineral concentration (Zn, Fe, Cu, Mn, Mg, Ca, P, and K), seed morphology phenotypes (seed area, seed eccentricity, thousand seed weight, and seed color), and agronomic phenotypes (grain yield, plant height, days to heading, days to maturity, and percent emergence). Varieties from the researcher-run trial showed significant differences for all traits excluding percent emergence. Samples from producer-run trials showed differences by location for concentration of all minerals and for all seed morphology traits but were not analyzed for agronomic phenotypes. Samples from producer-run trials showed no difference by variety for mineral concentration but showed varietal differences for all seed morphology phenotypes. Most minerals were positively correlated with one another (0.28 < *r* < 0.92). Grain yield was negatively correlated with Zn (*r* = −0.55, *p* < 0.01) and was positively correlated with plant height (*r* = 0.62, *p* < 0.001), seed area (*r* = 0.45, *p* < 0.05), and thousand seed weight (*r* = 0.45, *p* < 0.05). Results from this study can inform variety selection for stakeholders interested in adopting proso millet in the inland PNW and can support future proso millet breeding efforts, particularly in this region.

## 1 Introduction

Climate change increases abiotic and biotic stressors that will challenge the resiliency of agricultural production systems around the world (1). At the same time that these stressors are becoming more severe, our global population continues to rise, creating a mounting global demand for nutrient-dense calories (2–4). In order to meet this demand in the wake of climate change, greater resiliency must be cultivated in industrial agricultural systems. One promising strategy to increase resiliency is by increasing crop diversity within these systems. Just three major cereal crops, wheat (*Triticum aestivum* L.), maize (*Zea mays* L.), and rice (*Oryza sativa* L.), are estimated to sustain 50% of the caloric demand of the world population (2). However, there are a number of alternative crops including millets, quinoa, buckwheat, and amaranth with promising nutritional profiles and climate resilient traits that have not been as thoroughly developed for agricultural intensification and are currently being underutilized in certain parts of the world (5). Limitations exist in these crops regarding yield potential, trait improvement, knowledge diffusion, and market buy-in, stymieing their potential for adoption (6). Furthermore, solutions to these limitations, including management strategies, breeding for crop improvement, and market development, are often dependent on the specific region where adoption is being considered.

As researchers endeavor to improve agronomic and end-use qualities of under-researched crops, they may find a range of motivations for varietal selection and breeding goals from different actors across the food system. Analysis of mineral characterization and seed morphology, when considered in concert with agronomic phenotypes, can help inform variety selection for the whole food system, including processors and consumers, rather than narrowly focusing in on increased yields in the field.

The inland Pacific Northwest (PNW) is a semiarid region including Central Washington, Northeast Oregon, and Northern Idaho that is dominated by dryland cereal production (7, 8). More specifically, rain-fed wheat-fallow cropping systems are pervasive across the landscape, with lesser quantities of other small grains, legumes, and canola incorporated into rotations (7). The region is characterized by cold, wet winters and hot, dry summers, though average temperature and average precipitation (between 180 mm to 1,130 mm) depend on elevation and local topography (9).

Considering the climactic characteristics of the region and existing cropping systems, one crop with potential to increase the diversity and resiliency of the inland PNW is proso millet (*Panicum miliacium* L.) (10). Millets are small-seeded cereal crops that grow in semi-arid environments and have gained interest as climate resilient grains as they are highly water efficient, can grow on shallow, low fertile soils with a high range of salinity and acidity, and are C4 crops meaning that they take up more carbon dioxide from the environment than wheat and rice (5). Additionally, millets have a comparable nutritional profile to other major cereal grains, making them a promising crop for helping to improve food security in the wake of climate change (11). Millets are currently a staple food source for millions of people in arid and semiarid regions of India, Africa, and China, but in an effort to raise awareness and stimulate research and development of these grains in other parts of the world, the United Nations declared 2023 “International Year of the Millets” (12, 13).

There are approximately 20 different species of millets grown around the world for food, feed, forage, and fuel, that vary greatly in plant and seed morphology (14). However, proso millet is the species of greatest interest for human consumption in the US (15). Production and development of proso millet varieties have been historically concentrated in the Central Great Plains of Nebraska, Colorado, and South Dakota, and despite desirable nutritional characteristics, have been largely siloed into the birdseed market (14). Fourteen cultivars of proso millet have been developed in the US since the 1960s, and the six most commonly cultivated varieties were developed at the University of Nebraska-Lincoln, which houses the only proso millet breeding program in the country (16). North American cultivars have a narrow genetic base because of a limited number of parents in breeding (16).

Agronomic qualities and environmental requirements of these Midwest varieties show potential compatibility with production systems in the inland PNW (10, 14, 17). Proso millet is compatible with the winter wheat rotations characteristic of the region, as it is typically planted in late May or early June, and with a short growing season of 60–100 days can be used either as a replacement for summer fallow or as an emergency crop if an earlier seeded crop were to fail (18, 19). Proso millet has been shown to benefit these rotations and increase winter wheat yields by controlling winter annual grassy weeds, reducing insect and disease pressures, and preserving soil moisture (20). It also fills a unique niche for producers in the region looking for ways to diversify their rotations, as there are not currently any other warm season grasses commonly cultivated in the area. Finally, proso millet is well-adapted to the rainfed, dryland cropping systems characteristic of the region, as well as its well-drained loamy soils (10).

In this study we evaluated agronomic, nutritional, and seed morphology phenotypes of seven proso millet varieties grown in the inland Pacific Northwest. Varieties included “Dawn,” “Earlybird,” “Horizon,” “Huntsman,” “Plateau,” “Sunrise,” and “Sunup.” The overall goal of the study was to assess agronomic, nutritional, and seed morphology traits of commercially available proso millet varieties grown in the inland Pacific Northwest to better understand their potential for adoption into the regional food system. Specific objectives were to: (1) evaluate differences in zinc, iron, copper, manganese, magnesium, calcium, and phosphorus concentration in each variety; (2) compare the area, eccentricity, color, and thousand seed weight of seed from each variety; and (3) compare yield, plant height, days to heading, days to maturity, and percent emergence of each variety in this environment.

## 2 Materials and methods

### 2.1 Location

#### 2.1.1 Researcher-led trial

Samples for all research activities were collected from a single-year, researcher-run trial conducted in 2022 at Spillman Agronomy Farm in Pullman, WA (46.69743 °N Lat., −117.14720 °W Long.). Meteorological data were obtained from Pullman meteorological station located at 46.7 N Lat., −117.15 W Long, and elevation 760 m. Pullman received a total of 522 mm of total precipitation in 2022 and the average temperature was 8.3°C (21) (Table 1). The growing season was preceded by an uncharacteristically cold and wet spring, recorded at Washington state’s third coldest June on record and above average precipitation in April, May, and June 2022 (22). In contrast, Washington also experienced its hottest average temperature for the month of August in the same growing season of 2022 (22).

**Table 1:** Total precipitation (TP) and average maximum day temperature (MT) recorded during the growing season (May to September 2022) in Pullman, WA.

Year	Month	TP [mm]	AT [°C]
2022	June	93.98	20.5
	July	10.92	28.4
	August	0.51	31.2
	September	36.32	24.8

Meterological data were collected from Pullman, WA meteorological station situated at 46.7°N Lat., −117.15°W Long., and elevation 759.86 m. Source: WSU AgWeatherNet (21).

#### 2.1.2 Producer-led trials

Samples for seed morphology and mineral characteristics were collected from both the researcher-led trial in Pullman, WA, and a series of on-farm, producer-led trials across the region. One site was located in Edwall, WA (47.44474, −117.887), one in Mansfield, WA (47.91997, −119.795), and three in Genessee, ID (46.62175, −116.895; 46.56439, −116.831; 46.50229, −116.811) (Table 2). Elevation ranged from 713 m to 866 m and annual precipitation ranged from 6 to 22 in.

**Table 2:** Producer-run trial data including location (LOC), latitude (LAT), longitude (LONG), elevation (ELV in m), annual precipitation (AP) (in), planting date (PD), and harvest date (HD).

Trial	LOC	LAT	LONG	ELV	AP	PD	HD
1	Edwall, WA	47.44474	−117.887	713	11	6/12/2022	10/4/2022
2	Mansfield, WA	47.91997	−119.795	866	6–9	6/20/2022	9/27/2022
3	Genesee, ID	46.62175	−116.895	850	22	6/1/2022	10/15/2022
4	Genesee, ID	46.56439	−116.831	853	18–22	5/26/2022	9/28/2022
5	Genesee, ID	46.50229	−116.811	745	22	5/24/2022	9/13/2022

Elevation data source: USGS National Map Viewer, 2023; Annual precipitation data source: Self-reported from producer participants.

### 2.2 Plant materials

The 2022 trial at Spillman farm in Pullman, WA included seven test varieties (Table 3). These were selected as they were the only varieties that we were able to access commercially in the US in sufficient quantities. Dawn (23), Earlybird (24), Horizon (25), Huntsman (26), Plateau (27), and Sunrise (28) were sourced from Kriesel Seen Inc. in Gurley, Nebraska, and Sunup (29) was sourced from Perry Brothers Seed Inc. in Otis, Colorado. Dawn, Earlybird, Huntsman, Sunup, and Plateau were all developed by the Nebraska Agricultural Experiment Station (18). Horizon was developed by the Nebraska Agricultural Experiment Station in cooperation with the University of Wyoming, South Dakota State University, and the USDA-ARS Central Great Plains Research Station (25). Sunrise was released jointly by the Institute of Agriculture and Natural Resources, University of Nebraska, and USDA-ARS (28). Dawn is the earliest developed variety, released in 1975 and originally introduced as an experimental line from the Soviet Union, and is one of the parents of most varieties in the study (18, 23). Sunup was released in 1989 and the leading variety at the time. Earlybird, an early-maturing, short-stature variety, and Huntsman, a high-yielding late-maturing, tall variety, were both released in 1994, followed shortly thereafter by Sunrise, a large-seeded, high-yield variety released in 1995. Horizon was developed as an earlier-maturing, short stature variety in 2003. Most recently, Plateau, a cross between Huntsman and a Chinese waxy accession, was developed in 2014 for applications in food and industrial use (18, 27). Commercially available varieties were selected as they are accessible to growers who may be interested in adoption, but their performance has not been thoroughly assessed in the inland PNW environment.

**Table 3:** Proso millet variety used for variety trials to evaluate agronomic, mineral, and seed morphology phenotypes when grown in the inland Pacific Northwest in 2022.

Entry name	Seed source	Release date	Developer	Pedigree	Marketed traits
“Dawn”	KS	1976	NAES	Initially introduced as PI 260053 from the USSR	Early maturing Short Moderate yield Large seed size Compact panicle type
“Earlybird”	KS	1993	NAES	Selected from the cross “Minco”/NE76010//”RiseV” NE 79017; NE76010 was a selection from “Dawn”/”Panhandle” and NE79017 was a selection from Dawn/NE76010	Early maturing Short Good yield Large seed size
“Horizon”	KS	2003	NAES UoW SDSU CSU USDA-ARS	Single-plant F4 selection from bulk population including “Sunup,” “Rise,” “Dawn,” “Cope,” and three lines later released as “Earlybird,” “Sunrise,” and “Huntsman”	Early maturing Short
“Huntsman”	KS	1994	NAES USDA-ARS	Selected from the cross NE79012/NE79017/3/”Cope”// “Dawn”/”Common”; NE79012 is a selection from a Dawn/NE76004 cross and NE79017 is a selection from the cross Dawn/NE76010. NE76004 is a selection from a Dawn/”Min 402” cross and NE76010 is a selection from Dawn/"Panhandle”	Late maturing Tall Excellent yield Large seed size
“Plateau”	KS	2014	NAES	Cross “Huntsman”/PI 578074// PI 436626 (cataloged as “Lung Shu #18” in Germplasm Research Institute of China)	Waxy starch Medium height Moderate yield Good yield Small seed size
“Sunrise”	KS	1995	IANR, UNARD, USDA-ARS	Selected from the cross NE83014/NE83007, and has the expanded pedigree “Minn402V2*”"Dawn”// “Panhandle72”*Dawn/3/ “Minco”//Dawn/Panhandle	Mid-season maturing Good yield Large seed size Lodging tolerance
“Sunup"	PB	1989	NAES	Increase of an F4 derived proso line from the cross “Rise” X “Dawn”	Good yield (at time of release) Small seed size

### 2.3 Experimental design and data collection

#### 2.3.1 Researcher-led trial

Varieties were planted in a randomized complete-block design with four replicated blocks arranged in a grid format. Each plot was 7.4 m^2^ (80 ft^2^) split into 4 rows with 25.4 cm (10 in) between rows and one 76.2 cm (30 in) alley. Each row contained 2.4 g of seed to approximately represent a seeding rate of 19.1 kg/ha (17 lb./acre).

During the growing season, plots were routinely hand-weeded. The trial was not irrigated. No in-season fertilizer or herbicide was applied.

Plots were harvested by hand, cut with sickles at the base of the stem, bundled, and placed in a greenhouse for between 3 days and 2 weeks to facilitate drying of material. Hand harvesting helped prevent soil contamination of samples, reducing risk of mineral contamination. Plots were harvested over the course of 12 days based on maturity. Dried bundles from each plot were threshed using a Vogel thresher (Bill’s Welding, Pullman, WA, United States). Each sample of threshed seed was run through a 2021 Wintersteiger Classic Plus Plot Combine and tabletop sifter (Clipper Separation Technologies, Office Tester Seed Cleaner) to remove excess plant matter and other debris before yield weight was recorded.

#### 2.3.2 Producer-led trials

On-farm, producer-led trials were planted in unreplicated, side-by-side strips using full-scale planting equipment belonging to the producer. Producers were given the opportunity to plant between three to seven varieties depending on their capacity and were provided with 50 pounds of seed per variety. Varieties were randomly assigned to each producer participant, except Huntsman, which was included at each site as a control. The order in which varieties were planted was also randomly assigned by the research team. While researchers provided instruction on trial layout, all other planting and management decisions throughout the season were made by producer participants.

When each trial reached maturity, researchers conducted a site visit and collected subsamples from each variety strip. Strips were walked from one end to the other and a 1 m^2^ quadrat was used to hand-harvest harvest five subsamples distributed evenly throughout the strip. Similar to the Researcher-led trials, careful hand harvesting reduced the risk of soil contamination. Subsamples were cut with sickles at the base of the stem, bundled, and placed in a greenhouse for between 3 days and 2 weeks to facilitate drying of material. Dried bundles from each plot were threshed using a Vogel thresher (Bill’s Welding, Pullman, WA, United States). Each sample of threshed seed was run through a 2021 Wintersteiger Classic Plus Plot Combine and tabletop sifter (Clipper Separation Technologies, Office Tester Seed Cleaner) to remove excess plant matter and other debris. Further processing was conducted on subsamples for seed scanning and mineral analysis.

#### 2.3.3 Mineral phenotypes

Subsamples of seed from researcher-run and producer-run plots were collected and then further processed for mineral analysis. The hull was removed from whole seed using a household rice polishing machine (Takumuajiami White MB-RC52, Michiba Kitchen, Yamamato Electric, Fukushima, Japan) which separates seed from hull through the abrasion of a spinning, custom-made stainless-steel mesh basket. Stainless steel was intentionally used to reduce risk of mineral contamination. Hulls and broken seeds were then separated from samples using a series of stainless-steel sieves, and seeds that were not successfully dehulled through polishing were manually removed from each sample. Dehulled samples were then milled down into flour using an IKA A 10 Basic Mill (IKA Works Inc., Wilmington, NC, United States).

For each sample, 250 mg (+/− 5 mg) of flour was added to a 75 mL PTFE digestion vessel containing 2 mL DIW, and an additional 10 mL of DIW and 2 mL of HNO_3_ were added. Vessels were then capped and vortexed for 1 min in order to mix flour and acid, before an additional 2 mL of H_2_O_2_ was added. Caps were removed, and samples were pre-digested for 15 min. A Mars6 Xpress Microwave System (CEM Corporation, Matthews, NC, United States) with 40 PTFE vessel holders were used to digest each sample. Elemental analysis was then conducted using an Agilent MP-AES 4200 (Agilent Technologies, Santa Clara, CA, United States) equipped with a double pass glass cyclonic spray chamber, OneNeb V2 Nebulizer, and an SPS-3 autosampler (Agilent Technologies, Santa Clara, CA, United States).

#### 2.3.4 Seed morphology phenotypes

Subsamples for seed scanning were dehulled using a Tangential Abrasive Dehulling Device (Saskatoon, Sask., Canada).

Seed morphology data was generated with a system of flatbed scanners, using methods developed for comparable morphology analysis of quinoa (30). Two 1–2 g subsamples of clean seed were collected from each plot of the trial and distributed across the glass surface of a scanner and covered with a black background. Scanners then captured an 8-bit red, green, and blue (RGB) image at a resolution of 1,200 dots per inch (dpi) for each sample. These images were analyzed using the All Grains tool from the phytoMorph Image Phenomics Toolkit. This tool, developed by Moore et al. (31), generated average seed area, major axis (length), minor axis (width), and eccentricity (length:width ratio) in pixels. Seed area was converted from pixels squared to millimeters squared based on image resolution. The tool also generated a count of individual seeds in each image, using an approach originally developed for the analysis of maize kernels (32). The tool produced average values for the intensity of red, green, and blue (i.e., RGB) of each pixel within each seed (30). RGB decimal codes were generated by multiplying intensity averages by 255, creating a quantitative value corresponding to a specific color within the RGB color model. Principal Component Analysis was performed on RGB color space to reduce the three values to two latent factors. Thousand seed weight (TSW) was calculated with the weight of each sample divided by the algorithmically-counted seed number, multiplied by 1,000.

#### 2.3.5 Agronomic phenotypes

Percent emergence was estimated visually for each plot based on expected density of plants in each row. Heading was quantified by the number of days from planting to 50% heading. Plant length of five randomly selected individuals was measured from the base of the stem to the end of the panicle and mean length was recorded (102 days after planting in 2022). Maturity was determined when approximately 75% of plants had dry panicles and “ripe” seed (grain hard, difficult to divide with thumbnail) (19).

### 2.4 Statistical analysis

Statistical analyses were conducted using the R statistical software (33).

Levene’s test was conducted separately for researcher-run trial data and producer-run trial data for all outcomes. Analysis of variance (ANOVA) was conducted with function “aov” to determine if any of the given outcomes (mineral, morphological, and agronomic phenotypes) differed by variety for both trial groups.

Based on results from Levene’s test and ANOVA’s, further analysis was only conducted for researcher-run trial data. Effect size was calculated with function “etaSquared” from the “lsr” package. The package “LSD.test” from R package “agricolae” was used to produce means, coefficient of variation (C.V.), and least significant difference (LSD) values for each outcome. Finally, Pearson correlation analysis was performed with functions “cor” and “cor.test” to assess the relationship between all mineral, morphological, and agronomic traits for researcher-run trial samples. Statistical significant level was set at α = 0.05.

It should be noted that there may be discrepancies in significance indicated by groupings and LSD values. LSD calculations require an even dataset and groupings do not. Therefore, varieties with missing data points (uneven data sets) were eliminated for LSD calculations but were included for grouping calculations. Both are provided for reference.

## 3 Results

### 3.1 Mineral concentration

#### 3.1.1 Researcher-run trial

**Figure 1:**
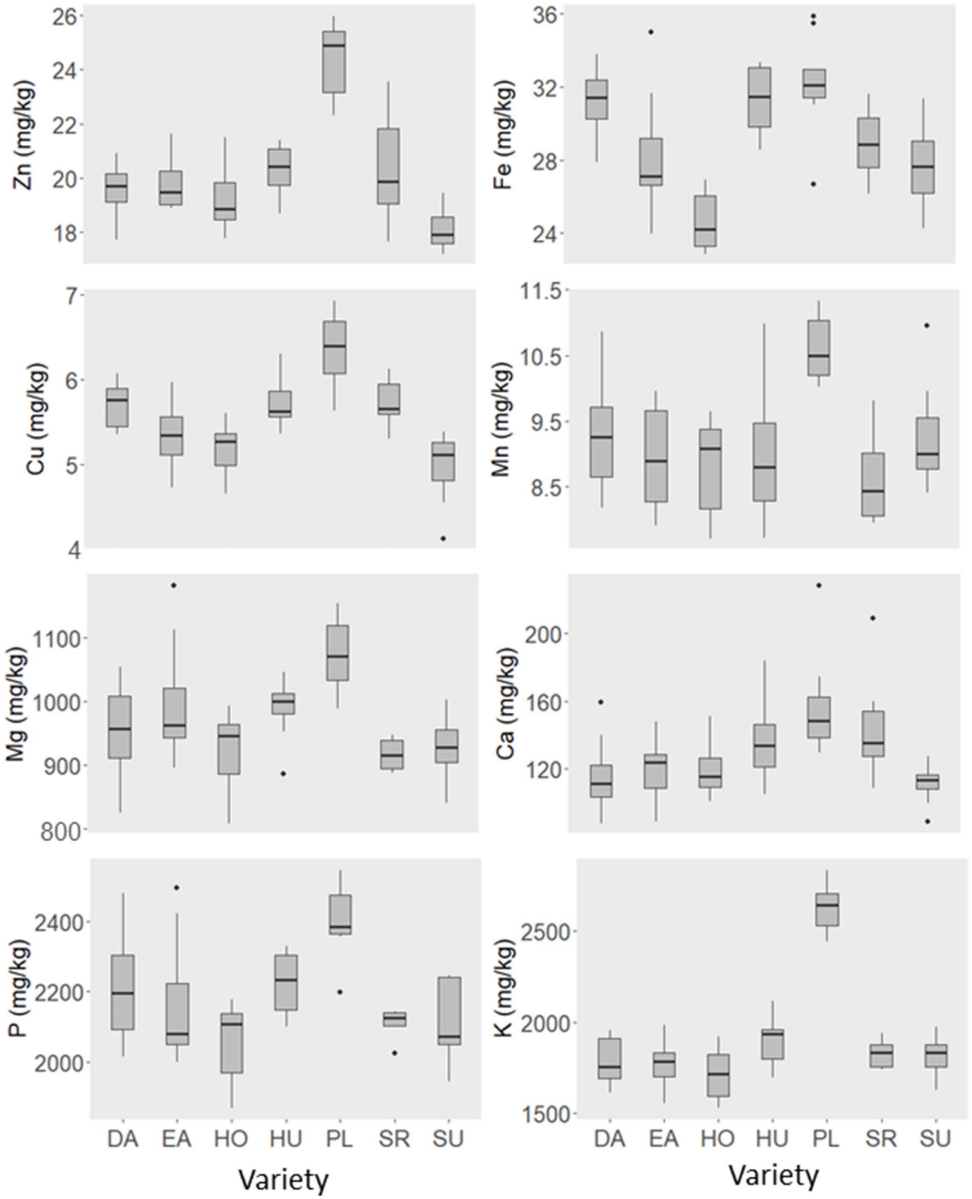
Distribution of proso millet mineral concentrations by variety from the 2022 researcher-run trial. Varieties: DA (Dawn), EA (Earlybird), HO (Horizon), HU (Huntsman), PL (Plateau), SR (Sunrise), SU (Sunup). Statistical differences between varieties can be found in Table 5.

Significant differences were found for all minerals by variety (Table 4). Plateau had a high concentration of every element, and was higher than all other varieties for Zn, Cu, Mn, Mg, P, and K (Table 5; Figure 1). Sunup had a lower concentration of Zn than other varieties and a lower concentration of Cu than all varieties but Horizon. Horizon had a lower concentration of Fe than other varieties. Mn was consistent across all varieties except Plateau, which had a higher concentration.

**Table 4:** Analysis of variance for mineral concentration (mg/kg) for proso millet varieties grown in researcher-run and producer-run trials in 2022.

		Df	Sum Sq	Mean Sq	*F*-value	Pr(>F)	η^2^	Significance
Zn								
Researcher	Variety	6	188.43	31.406	19.72	2.14e-11	0.72	***
	Residuals	47	74.84	1.592				
Producer	Location	4	424.8	106.20	13.681	1.31e-06		***
	Variety	6	96.2	16.03	2.065	0.0854		
	Residuals	32	248.4	7.76				
Fe								
Researcher	Variety	6	329.1	54.84	9.281	1.04e-06	0.54	***
	Residuals	47	277.7	5.91				
Producer	Location	4	4957	1239.2	64.202	7.86e-15		***
	Variety	6	87	14.5	0.752	0.613		
	Residuals	32	618	19.3				
Cu								
Researcher	Variety	6	9.887	1.648	12.02	3.88e-08	0.61	***
	Residuals	47	6.441	0.137				
Producer	Location	4	16.688	4.172	14.300	8.47e-07		***
	Variety	6	3.704	0.617	2.116	0.0787		
	Residuals	32	9.336	0.292				
Mn								
Researcher	Variety	6	19.6	3.266	4.968	0.000519	0.39	***
	Residuals	47	30.9	0.657				
Producer	Location	4	97.71	24.428	14.813	5.96e-07		***
	Variety	6	8.15	1.359	0.824	0.56		
	Residuals	32	52.77	1.649				
Mg								
Researcher	Variety	6	144,061	24,010	5.644	0.000178	0.42	***
	Residuals	47	199,932	4,254				
Producer	Location	4	283,187	70,797	3.705	0.0138		*
	Variety	6	60,235	10,039	0.525	0.7848		
	Residuals	32	611,499	19,109				
Ca								
Researcher	Variety	6	13,986	2330.9	4.04	0.00241	0.34	**
	Residuals	47	27,114	576.9				
Producer	Location	4	4,110	1027.5	1.366	0.267		**
	Variety	6	5,197	866.1	1.152	0.356		
	Residuals	32	24,066	752.1				
P								
Researcher	Variety	6	585,492	97,582	6.126	8.47e-05	0.44	***
	Residuals	47	748,636	15,928				
Producer	Location	4	1,536,371	384,093	5.677	0.00143		**
	Variety	6	408,119	68,020	1.005	0.43905		
	Residuals	32	2,164,982	67,656				
K								
Researcher	Variety	6	4,771,577	795,263	47.1	<2e-16	0.86	***
	Residuals	47	793,557	16,884				
Producer	Location	4	2,287,037	571,509	4.728	0.00412		**
	Variety	6	261,687	43,614	0.361	0.89816		
	Residuals	32	3,868,296	120,884				

Significant level at (*p* < 0.05) while *p < 0.05, **p < 0.01, and ***p < 0.001.

**Table 5:** Mean data of proso millet varieties for zinc (Zn), iron (Fe), copper (Cu), manganese (Mn), magnesium (Mg), calcium (Ca), phosphorus (P), and potassium (K), seed area (SA), seed eccentricity (SE), thousand seed weight (TSW), seed color (SC), grain yield (GY), plant height (PH), days to heading (DH), days to maturity (DM), and percent emergence (PE) from the 2022 researcher-run trial.

Variety	Zn (mg/kg)	Fe (mg/kg)	Cu (mg/kg)	Mn (mg/kg)	Mg (mg/kg)	Ca (mg/kg)	P (mg/kg)	K (mg/kg)	SA (mm^2^)	SE	TSW (g)	SC	GY (g/m^2^)	PH (cm)	DH (days)	DM (days)	PE (%)
Dawn	19.6 (b)	30.2 (ab)	5.71 (bc)	9.30 (b)	951 (bcd)	116 (cd)	2,215 (b)	1,788 (bc)	3.46 (a)	1.079 (d)	5.11 (a)	−21.70 (d)	749 (a)	132 (a)	67 (ab)	114 (a)	70 (ab)
Earlybird	19.8 (b)	28.3 (c)	5.36 (cd)	8.95 (b)	997 (b)	119 (cd)	2,165 (bc)	1,770 (bc)	3.44 (cd)	1.082 (bcd)	5.03 (ab)	−18.90 (c)	685 (ab)	127 (ab)	69 (a)	112 (ab)	65 (b)
Horizon	19.3 (bc)	24.6 (d)	5.18 (de)	8.84 (b)	922 (cd)	120 (bcd)	2,061 (c)	1,721 (c)	3.39 (bc)	1.087 (b)	5.10 (a)	−13.60 (b)	683 (ab)	127 (ab)	67 (ab)	112 (ab)	86 (a)
Huntsman	20.3 (b)	31.2 (ab)	5.75 (b)	9.02 (b)	987 (bc)	136 (abc)	2,224 (b)	1,894 (b)	3.33 (de)	1.080 (cd)	4.91 (b)	−19.06 (c)	679 (ab)	123 (abc)	67 (ab)	111 (abc)	90 (a)
Plateau	24.4 (a)	32.1 (a)	6.36 (a)	10.61 (a)	1071 (a)	158 (a)	2,400 (a)	2,626 (a)	3.04 (f)	1.095 (a)	4.17 (d)	−9.15 (a)	638 (abc)	121 (abc)	67 (ab)	108 (bcd)	85 (ab)
Sunrise	20.4 (b)	28.9 (bc)	5.73 (bc)	8.63 (b)	916 (d)	146 (ab)	2,111 (bc)	1827 (bc)	3.44 (ab)	1.087 (bc)	5.15 (a)	−15.92 (b)	599 (bc)	119 (bc)	64 (b)	106 (d)	82 (ab)
Sunup	18.1 (c)	27.5 (c)	4.97 (e)	9.23 (b)	927 (cd)	111 (d)	2,115 (bc)	1815 (bc)	3.30 (e)	1.080 (d)	4.75 (c)	−29.38 (e)	535 (c)	112 (c)	59 (c)	107 (cd)	75 (ab)
C.V. %	6.2	8.3	6.64	8.76	7	19	6	7	1.78	3.75	2.85	−14.15	11	6	5	3	17
LSD (0.05)	1.1*	2.5*	0.38*	0.83*	69*	22*	134*	137*	0.06	0.013	0.14**	2.59	110***	11	4	4	21
Mean	20.2	29.1	5.57	9.25	969	129	2,187	1,924	3.33	0.362	4.89	−18.24	653	123	66	110	79

Dissimilar letters in a column are significantly different at *p* < 0.05. LSD, Least Significant Difference; LSD comparisons are significant at *p* < 0.05.

* LSD for mineral concentration excludes entries of “Sunrise” (*n* = 3) due to one missing entry. All other varieties contain four entries (*n* = 4) and LSD calculation requires an even dataset. Groupings were calculated with all data points, including “Sunrise” entries.

** LSD for TSW excludes “Sunrise” (*n* = 3) and “Sunup” (*n* = 3) due to two missing data entries. All other varieties contain four entries (*n* = 4) and LSD calculation requires an even dataset. Groupings were calculated with all data points, including “Sunrise” and “Sunup” entries.

*** LSD for GY excludes entries of “Dawn” (*n* = 3) due to one missing entry. All other varieties contain four entries (*n* = 4) and LSD calculation requires an even dataset. Groupings were calculated with all data points, including “Dawn” entries.

#### 3.1.2 Producer-run trial

Two samples were removed (Location 2: Sunrise, Location 3 Huntsman) due to suspected soil contamination. Two-way ANOVA was conducted, with variety and location as independent variables. Location had a significant effect on all elements (Table 4). ANOVA was also conducted for the interaction of trial and variety but did not produce significant results for any elements. Sample size was low, as each location planted a single replicate of a subset of varieties. Means are provided for each location by variety (Table 6); further statistics were not conducted due to limited data. Analysis for each sample was replicated.

**Table 6:** Mean zinc (Zn), iron (Fe), copper (Cu), manganese (Mn), magnesium (Mg), calcium (Ca), phosphorus (P), potassium (K), seed area (SA), seed eccentricity (SE), thousand seed weight (TSW), and seed color (SC) from the 2022 producer-run trials.

	n=	Variety	Zn (mg/kg)	Fe (mg/kg)	Cu (mg/kg)	Mn (mg/kg)	Mg (mg/kg)	Ca (mg/kg)	P (mg/kg)	K (mg/kg)	SA (mm2)	SE	TSW (g)	SC
Location 1	2	Dawn	25.0	32.9	4.86	7.96	1,082	142	2,515	1,994	3.43	1.071	4.92	−22.17
	2	Earlybird	24.6	33.0	4.99	8.94	1,239	163	2,606	2,356	3.07	1.097	4.23	−42.36
	2	Horizon	26.9	25.6	3.74	7.37	1,051	133	2,375	2,365	2.95	1.107	4.08	−48.77
	2	Huntsman	25.3	31.7	4.75	8.83	1,228	140	2,716	2,992	2.98	1.092	4.00	−48.45
	2	Plateau	22.6	28.1	4.66	7.99	973	190	2,191	2,587	2.92	1.107	3.84	−28.54
	2	Sunrise	20.5	26.6	4.33	8.72	1,003	148	2,235	2,601	3.17	1.094	4.46	−33.57
	2	Sunup	28.0	27.6	4.08	7.69	1,137	151	2,718	2,726	2.98	1.118	3.98	−39.19
Location 2	2	Horizon	30.9	73.6	4.61	15.85	1,359	149	3,130	2,650	3.28	1.098	4.71	−43.73
	2	Huntsman	29.1	62.5	5.19	11.25	1,339	138	3,107	2,867	3.22	1.092	4.64	−42.20
Location 3	2	Dawn	22.9	38.4	3.46	9.21	1,278	118	3,130	2,519	3.68	1.061	5.45	0.20
	2	Horizon	21.7	33.6	3.29	6.94	1,025	137	2,328	2,445	3.72	1.069	5.82	−5.07
	2	Plateau	16.4	35.8	3.87	8.49	1,121	127	2,625	2,355	3.21	1.073	4.61	−20.20
	2	Sunup	22.2	30.1	4.23	8.41	1,076	137	2,520	2,322	3.50	1.046	5.07	3.86
Location 4	2	Earlybird	25.1	32.8	4.92	8.76	1,130	118	2,659	2,148	3.57	1.081	5.30	−8.13
	2	Huntsman	25.5	34.5	4.82	8.90	1,093	152	2,606	2,027	3.66	1.068	5.42	−8.92
	2	Plateau	25.5	32.0	5.23	8.96	1,107	141	2,611	2,229	3.24	1.074	4.49	−19.83
	2	Sunrise	27.1	33.9	5.41	9.27	1,166	104	2,770	2,248	3.63	1.078	5.38	−9.27
Location 5	2	Dawn	28.9	30.2	5.24	8.54	1,028	106	2,485	2,036	3.60	1.070	5.04	−7.90
	2	Huntsman	27.3	27.4	4.81	6.65	821	128	2,049	1,864				
	2	Plateau	31.3	33.6	6.66	10.14	1,175	157	2,756	2,129	3.15	1.079	4.24	−24.63
	2	Sunrise	27.8	33.3	5.92	8.40	1,087	133	2,496	2,014	3.57	1.073	5.30	−16.93

### 3.2 Seed morphology phenotypes

#### 3.2.1 Researcher-run trial

**Figure 2:**
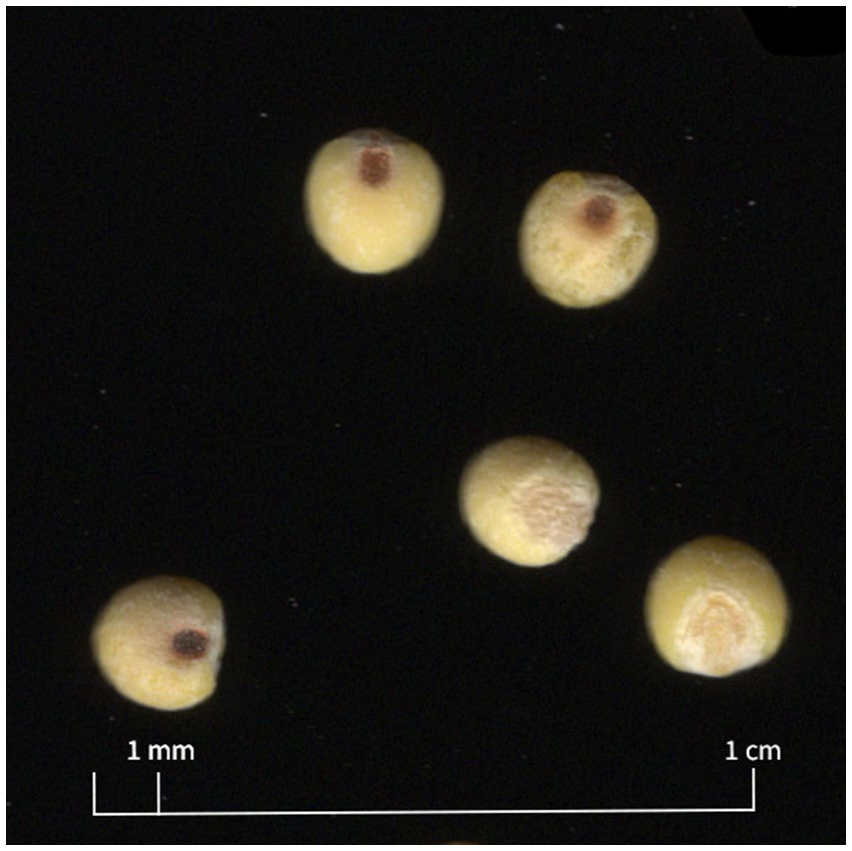
Close-up of seeds scan images (cropped from larger image) show examples of dehulled proso millet seed from 2022 researcher-run trial. Plateau (the smallest variety, lightest in color) is shown on the left **(A)** and Dawn (largest variety, darker in color) is shown on the right **(B)**. Some seeds show some signs of abrasion from the dehulling process.

There was a significant difference in seed area by variety (Table 7). Dawn was larger than all varieties except for Sunrise and Plateau was the smallest variety (Table 5; Figure 2). There was a significant difference in seed eccentricity by variety (Table 7). The length:width ratio of Plateau was furthest from 1, indicating that it was the least round (Table 5). There was a significant difference in thousand seed weight by variety (Table 7). Plateau weighed less than all other varieties, coinciding with its smaller area (Table 5). Varietal means for TSW ranged from 4.17 to 5.15 g (Table 5). There was a significant difference in seed color by variety (Table 7). Principle component analysis was performed to compare red, green, and blue values with a single value. Plateau has a higher PC value, indicating a lighter color. Sunup had the lowest PC value, indicating darker color than the other varieties (Table 5).

**Table 7:** Analysis of variance for seed morphology traits seed area (SA), seed eccentricity (SE), thousand seed weight (TSW), and seed color (SC) for proso millet varieties grown in researcher-run and producer-run trials.

		Df	Sum Sq	Mean Sq	*F*-value	Pr(>F)	η^2^	Significance
SA								
Researcher	Entries	6	4835319	805,886	46.19	<2e-16	0.85	***
	Residuals	49	854977	17,449				
Producer	Location	4	8242708	2,060,677	48.16	7.16e-13		***
	Variety	6	4714380	785,730	18.36	5.67e-09		***
	Residuals	31	1326389	42,787				
SE								
Researcher	Entries	6	0.001667	2.779e-04	5.559	0.000186	0.41	***
	Residuals	49	0.002450	4.999e-05				
Producer	Location	4	0.008092	0.0020230	21.787	1.22e-08		***
	Variety	6	0.001473	0.0002455	2.644	0.0345		*
	Residuals	31	0.002879	0.0000929				
TSW								
Researcher	Entries	6	5.590	0.9317	47.9	<2e-16	0.86	***
	Residuals	47	0.914	0.0194				
Producer	Location	4	7.220	1.8049	43.33	2.86e-12		***
	Variety	6	4.418	0.7363	17.68	8.84e-09		***
	Residuals	31	1.291	0.0417				
SC								
Researcher	Entries	6	1974.0	329.0	49.37	<2e-16	0.86	***
	Residuals	49	326.5	6.7				
Producer	Location	4	9271	2317.8	55.615	1.05e-13		***
	Variety	6	1111	185.1	4.442	0.00236		**
	Residuals	31	1292	41.7				

Significant level at (*p* < 0.05) while *p < 0.05, **p < 0.01, and ***p < 0.001.

#### 3.2.2 Producer-run trial

Differences were found for seed area, seed eccentricity, thousand seed weight, and seed color by location and by variety in the producer-run trials when two-way anova was run on producer-run trial data (Table 7). A one-way anova did not show differences in these traits for the producer-run trial. Means are provided for each location by variety (Table 6), however additional statistics were not conducted due to low sample size. Analysis for each sample was replicated. Location 5: Huntsman is missing due to sample loss. Principle component analysis was performed to compare red, green, and blue values with a single value.

### 3.3 Agronomic phenotypes (researcher-run trial)

There was a significant difference in grain yield by variety (Table 8). Sunup yielded more than Dawn and Plateau (Table 5). Plateau also yielded less than Huntsman, Sunrise, and Earlybird. Mean grain yield was 653 g/m^2^ with a least significant difference of 110. However, since LSD requires a balanced data set for calculation, and one of four samples of Dawn was missing, LSD was calculated with all Dawn samples excluded.

**Table 8:** Analysis of variance for grain yield (GY), plant height (PH), days to heading (DH), days to maturity (DM), and percent emergence (PE) for proso millet varieties grown in Pullman, WA in 2022.

	Df	Sum Sq	Mean Sq	*F*-value	Pr(>F)	η^2^	Significance
GY							
Entries	6	111,922	18,654	3.198	0.0229	0.49	*
Residuals	20^1^	116,675	5,834				
PH							
Entries	6	992.8	165.47	2.9	0.0321	0.45	*
Residuals	21	1198.3	57.06				
DH							
Entries	6	240.4	40.07	4.573	0.00406	0.57	**
Residuals	21	21	184.0	8.76			
DM							
Entries	6	207.4	34.56	3.845	0.0096	0.52	**
Residuals	21	188.8	8.99				
PE							
Entries	6	2016	336.0	1.783	0.151	0.37	
Residuals	21	3957	188.4				

Significant level at (*p* < 0.05) while *p < 0.05, **p < 0.01, and ***p < 0.001.

1 One observation deleted due to missingness (missing data for one observation of “Dawn”).

There was a significant difference in plant height by variety (Table 8). Sunup was taller than Dawn and Plateau (Table 5). Huntsman and Sunrise were also taller than Plateau.

There was a significant difference in days to heading by variety (Table 8). Plateau headed earlier than all other varieties (Table 5). Dawn headed earlier than Huntsman.

There was a significant difference in days to maturity by variety (Table 8). Dawn matured more quickly than Sunup, Huntsman, Sunrise, and Earlybird (Table 5). Plateau and Horizon also matured more quickly than Sunup.

There was no significant difference in percent emergence by variety. Effect size calculation suggests that 34% of percent emergence can be explained by variety, which is lower than effect size for other agronomic outcomes in the study, but still very large (Cohen’s *f* = 0.71) (34) (Table 8). Sample size may have been too small to produce significant results at this effect size. Further research with a larger sample size could be conducted to clarify results.

### 3.4 Correlation

#### 3.4.1 Mineral concentration

All minerals are positively correlated with one another except for Fe and Ca (Table 9). Correlations range from *r* = 0.28 to *r* = 0.92, with highest correlations (*r* > 0.7) between Zn and K, Fe and Mg, Fe and P, Mn and Mg, Mn and P, and Mg and P.

**Table 9:** Pearson correlation for all phenotypes: grain yield (GY), plant height (PH), days to heading (DH), days to maturity (DM), percent emergence (PE), seed area (SA), seed eccentricity (SE), seed color (SC), thousand seed weight (TSW), zinc (Zn), iron (Fe), copper (Cu), manganese (Mn), magnesium (Mg), calcium (C), phosphorus (P), and potassium (K) from the 2022 researcher-run trial.

	GY	PH	DH	DM	PE	SA	SE	SC	TSW	Zn	Fe	Cu	Mn	Mg	Ca	P
PH	0.62 ***															
DH	0.24	0.21														
DM	0.3	0.35	0.55 **													
PE	0.11	0	−0.21	−0.4 *												
SA	0.45 *	0.57 **	0.43 *	0.12	0.27											
SE	−0.54 **	−0.34	−0.36	−0.16	0.25	−0.53 ***										
SC	−0.48 *	−0.51 **	−0.42 *	−0.43 *	0.28	−0.4 **	0.67 ***									
TSW	0.45 *	0.51 **	0.45 *	0.12	0.29	0.98 ***	−0.48 ***	−0.33 *								
Zn	−0.55 **	−0.67 ***	−0.57 **	−0.4 *	−0.06	−0.63 ***	0.55 ***	0.59 ***	−0.62 ***							
Fe	−0.13	−0.11	−0.36	−0.21	−0.29	−0.28 *	0.08	0.14	−0.31 *	0.51 ***						
Cu	−0.36	−0.41 *	−0.53 **	−0.37	−0.18	−0.39 **	0.31 *	0.47 ***	−0.45 ***	0.68 ***	0.67 ***					
Mn	−0.18	0.06	−0.52 **	−0.25	−0.2	−0.43 **	0.31 *	0.25	−0.48 ***	0.28 *	0.47 ***	0.49 ***				
Mg	0	−0.09	−0.45 *	−0.24	−0.21	−0.43 **	0.25	0.37 **	−0.4 **	0.49 ***	0.71 ***	0.64 ***	0.76 ***			
Ca	0.03	−0.02	−0.3	0.07	−0.27	−0.28 *	0.22	0.37 **	−0.27	0.31 *	0.2	0.58 ***	0.3 *	0.37 **		
P	−0.2	−0.1	−0.43 *	−0.22	−0.37	−0.44 ***	0.24	0.3 *	−0.44 **	0.51 ***	0.77 ***	0.69 ***	0.79 ***	0.92 ***	0.38 **	
K	−0.41 *	−0.45 *	−0.66 ***	−0.28	−0.23	−0.82 ***	0.54 ***	0.51 ***	−0.83 ***	0.73 ***	0.37 **	0.66 ***	0.53 ***	0.53 ***	0.57 ***	0.6 ***

*p < 0.05, **p < 0.01, ***p < 0.001.

#### 3.4.2 Seed morphology

Seed color is highly correlated with seed eccentricity (*r* = 0.67, *p* < 0.001) (Table 9). Seed eccentricity is negatively correlated with seed area (*r* = −0.53, *p* < 0.001) and thousand seed weight (*r* = −0.48, *p* < 0.001), indicating that larger seeds are more round. As expected, seed area is extremely highly correlated with thousand seed weight (*r* = 0.98, *p* < 0.001), indicating that larger seeds weigh more.

#### 3.4.3 Agronomic phenotypes

Grain yield and plant height were highly correlated (*r* = 0.62, *p* < 0.001) (Table 9). Days to heading and days to maturity were also highly correlated (*r* = 0.55, *p* < 0.01). Percent emergence and days to maturity were moderately negatively correlated (*r* = −0.40, *p* < 0.05), suggesting that the best emergers were also quickest to mature, which is desirable as producers are looking for early-maturing varieties and good emergence.

#### 3.4.4 Correlations across mineral concentration, seed morphology, and agronomic phenotypes

Several agronomic traits were correlated with mineral content. Zinc was negatively correlated with plant height (*r* = −0.67, *p* < 0.001), days to heading (*r* = −0.57, *p* < 0.01), grain yield (*r* = −0.55, *p* < 0.01), and days to maturity (*r* = −0.4, *p* < 0.05) (Table 9). Days to heading was negatively correlated with all minerals except for Fe and *Ca.* Plant height was negatively correlated with zinc (*r* = −0.67, *p* < 0.001), potassium (*r* = −0.45, *p* < 0.05), and copper (*r* = −0.41, *p* < 0.05).

Days to heading was negatively correlated with all minerals besides Fe and *Ca.*

Seed morphology traits showed both negative and positive correlations with mineral content. Zinc and potassium showed correlations across all seed morphology traits. All minerals besides Ca showed some degree of negative correlation with both seed area and thousand seed weight, indicating that larger seed samples have lower mineral concentration. Strongest negative correlations with seed area include K (*r* = −0.82, *p* < 0.001) and Zn (*r* = −0.63, *p* < 0.001). Thousand seed weight, which coincides with seed area, also showed strongest correlation with K (*r* = −0.83, *p* < 0.001) and Zn (*r* = −0.62, *p* < 0.001).

All minerals besides Fe and Mn were positively correlated with seed color. Seed color was most strongly correlated with zinc (*r* = 0.59, *p* < 0.001) and potassium (*r* = 0.51, *p* < 0.001). Seed eccentricity was most highly correlated with zinc (*r* = 0.55, *p* < 0.001) and potassium (*r* = 0.54, *p* < 0.001).

No agronomic and seed morphology traits were correlated at a rate higher than *r* = 0.57, however plant height showed correlation with seed area (*r* = 0.57, *p* < 0.01), thousand seed weight (*r* = 0.51, *p* < 0.01), and negative correlation with seed color (*r* = −0.51, *p* < 0.01). Grain yield was negatively correlated with seed eccentricity (*r* = −0.54, *p* < 0.01). Grain yield was moderately positively correlated with TSW (*r* = 0.45, *p* < 0.05) and seed area (*r* = 0.45, *p* < 0.05).

## 4 Discussion

### 4.1 Mineral concentration

#### 4.1.1 Researcher-run trial

Based on this study, Plateau stood out as a mineral-rich variety of proso millet, with high concentration of all tested minerals compared to the other test varieties. Plateau is the only test variety that was developed for waxy starch end-use quality, bred using a waxy Chinese accession as a parent, which could contribute to its high mineral concentration (18, 27).

Macronutrients, such as K, Ca, P, and Mg, and micronutrients such as Zn, Cu, Fe, and Mn all serve important roles in human nutrition. While deficiency of macronutrients can result in hunger, wasting, and stunted growth, micronutrient deficiencies have less detectable physical manifestations, and are therefore easier to overlook (35). Approximately three billion people worldwide suffer from micronutrient deficiencies (36).

Studies have been more frequently conducted on the macronutrients and micronutrients of pearl millet than proso millet, but some studies compare proso millet mineral concentration with other millets and other grains (35, 37–40).

In a comparison of foxtail millet, little millet, barnyard millet, kodo millet, finger millet, and sorghum, with three Indian cultivars of proso millet, foxtail and barnyard millet had less Fe than proso millet while little millet and barnyard millet had higher Zn (40). Finger millet and kodo had higher Mn than proso millet and all millets (excluding kodo) had more P than proso millet (40). All small millets, including proso millet, had higher Zn, Fe, K, Mn, Mg, and Cu than sorghum (40). Mean mineral concentration for proso millet in this study was higher for all tested elements (Ca, P, K, Mg, Fe, Cu, Zn, Mn) than varieties in our study.

Proso millet has been shown to have higher concentrations of Mg, Fe, Mn, and Zn than rice, comparable levels of P, Mg, Fe, and Zn to maize, and lower levels of Ca, P, Fe, Mn, and Zn than wheat (11, 41). A study of Turkish cereal grains shows proso millet as also having a higher concentration of Ca and P than spring and winter wheat (38).

While different cultivars of a crop can vary in their macronutrient and micronutrient profiles, mineral concentration in crops has been shown to be linked to soil organic matter and management practices (42, 43). A Polish study comparing mineral concentration of proso millet in conventional and organic systems showed higher concentration of Cu, Mn, Fe, and Zn in proso millet produced organically, suggesting that production practices may influence mineral content for these elements regardless of variety (42). Mean results from conventional samples from this study, which used a proso millet variety “Jagna,” fell within variety averages from our study for Mg, Cu, and Zn while mean results were higher than variety averages from our study for Ca, Mn, and Fe (42).

#### 4.1.2 Producer-run trial

We saw differences in mineral concentration by location, however, as each producer-run trial location contained a different subset of varieties, had different baseline conditions (such as soil properties), and received different treatments (such as fertilizer application), we are unable to isolate what variable within “location” led to significant differences in these elements.

### 4.2 Seed morphology phenotypes

#### 4.2.1 Researcher-run trial

While differences in seed morphology traits were statistically significant, differences are visually negligible, and may be small enough to be insignificant for processing applications. For example, least significant difference for seed area is 0.06 mm^2^, and mean difference between the smallest variety (Plateau) and the largest variety (Dawn), is 0.42 mm^2^, or a difference in seed radius of approximately 0.07 mm (Table 5; Figure 2).

Regarding seed color, varieties in this study were “white” proso millet cultivars, ranging in shades of straw or light brown. While varieties did differ in color, there was not a highly detectable visual range of colors between varieties (Figure 2). However world core collections are also made up of accessions with light red, dark olive green, dark red, olive green, dark brown, dark green, brown, and black (44, 45). One study showed about 80% of accessions to be light brown, straw, or white (44) while a later study showed about 50% of *miliaceum* accessions to be in this group (45).

TSW results from this study (4.17–5.15 g) fell within the range from an analysis of the proso millet world core collection, of 3.9–6.6 g (45). Compared to other grains planted in the region, proso millet is more similar in weight to canola, which typically ranges from 2 to 6 mg seed^−1^, than wheat seed planted in the inland Pacific Northwest, which typically ranges from 31 to 38 mg seed^−1^ (46).

#### 4.2.2 Producer-run trial

While we did see differences in seed morphology traits by location, each of these locations received different treatments (environmental conditions, planting and harvest date, fertilizer, chemical dessication etc.). Differences cannot be attributed to any specific treatment but do suggest that traits can be affected by treatment.

### 4.3 Agronomic phenotypes (researcher-run trial)

Sunup was high yielding in our study, which was unexpected as Huntsman, Earlybird, Horizon, and Sunrise were all bred as high-yielding replacements for this older variety (18). In a meta-analysis of dryland proso millet variety trials from Sidney, NB, Akron, CO, and Lingle, WY between 2002 and 2013, Sunup yielded more than Dawn on average, which is consistent with our results (27). However, this same analysis showed Plateau yielding more than Sunup, contrary to our results. A 2017 proso millet trial in Musanze, Rwanda showed Sunup as lower yielding than Huntsman but higher yielding than Earlybird (47). Habiyaremye et al. (17), conducted an irrigated proso millet trial in Pullman, WA. Results cannot be directly compared as this study did not include Plateau or Dawn, but it showed Sunup as yielding more than Huntsman and Sunrise in 2012, more than Sunrise in 2013, and less than Huntsman and Sunrise in 2014 (17).

Sunup and Huntsman, two of the taller varieties in the study, were released after Dawn and marketed for their greater height, which increases potential for direct harvest using a combine equipped with a stripper-header (18). While increased plant height can improve harvest to an extent, too much height, greater than 150 cm according to Zhang et al. (48), can increase susceptibility to lodging. Mean plant height in the 2022 Pullman, WA trial was 123 cm and maximum plant height across varieties was 140 cm (Sunup), which did not exceed this upper limit (48). Consistent with the findings in our study, Santra et al. (27) found Sunup and Huntsman to be taller than Plateau in dryland trials in NB, CO, and WY.

A 2007 study of the world’s core collection of proso millet showed a range in mean plant height from 33 to 92 cm (44), which is lower than the range of means in our study (112–132 cm), however a later core collection study found mean plant height ranging from 64 to 175 cm which includes the range of our results (48).

While Plateau headed earlier than other varieties in our study, it did not form heads earlier than other varieties in Midwest trials (27). If proso millet were being grown as a forage crop, it would need to be harvested soon after heading to optimize forage quality (18). Analysis of the global proso millet germplasm collection found a high Shannon-Weaver diversity index (H′) value for days to 50% flowering, indicating opportunity for breeding for fewer days to heading (45).

Early maturity is a desirable trait for producers in the inland PNW who need to harvest their crop before the rainy fall season begins. The 2022 researcher-run trial was harvested on a plot-by-plot basis as they reached maturity, however all remaining plots had to be harvested on day 113 after planting, regardless of if full maturity had been reached. These samples (*n* = 5) were logged as DM = 114, which could have slightly altered means by variety for DM. However, proso millet is frequently swathed or chemically desiccated before full maturity is reached in order to expedite harvest, which was simulated in early harvest of these five samples (18).

### 4.4 Correlation

#### 4.4.1 Mineral concentration

We did not see negative correlation of any minerals, suggesting that breeders can seek to increase mineral concentration of select minerals without hampering concentration of others.

Zn and Fe were shown to be strongly positively correlated in studies of hard winter wheat, and were correlated in our study (*r* = 0.52, *p* < 0.01) (49, 50). Zn and Fe were also highly correlated in two studies of pearl millet (37, 51). One of these studies also showed strong correlation with Zn and Cu in pearl millet, which we observed in our study (51). Our study is consistent with the later of these two pearl millet studies, which showed a significant positive correlation between Fe and Cu, while the earlier study did not (37, 51). In one study of hard winter wheat, correlation of phosphorus was >0.05 with Mg, K, Fe, and Zn (49). This is consistent with our results. Strong positive correlations of Zn, Fe, and Cu, observed in our study and in the studies of other grains, reflect the underlying physiology that links the accumulation of Zn, Fe, and Cu in grain (49).

#### 4.4.2 Agronomic phenotypes

Negative correlation between percent emergence and days to maturity suggests that the best emergers were also quickest to mature. This is desirable as producers are looking for early-maturing varieties and good emergence.

Similar to our results, other studies of proso millet also found a positive correlation between grain yield and plant height, suggesting that plant height can be used for simple selection (52–54). Plant height has also been associated with rate of maturity, but we did not see this in our study (52). A study of pearl millet also found positive correlation for grain yield with plant height and thousand seed weight, which we saw in our study (37). Risk of lodging should be taken into consideration when selecting for plants with greater height.

#### 4.4.3 Correlations across mineral concentration, seed morphology, and agronomic phenotypes

While limited correlative studies have been conducted on proso millet’s agronomic and mineral phenotypes, a study of pearl millet found positive correlation of grain yield with Cu and Mn, and found genotypic, but not phenotypic, correlation with Fe (37). Grain yield was not correlated with Cu and Mn in our study.

Studies on mineral concentration of wheat have shown negative correlation between grain yield and Zn, which we observed in this proso millet study (49, 55–57). However, decreasing trends in concentration of Cu, Fe, and Mg have also been observed in high yielding varieties, which we did not observe in our study (49, 55). Lower mineral varieties in wheat also correspond to release date, dropping off significantly in the late 1960s when semi-dwarf, high yielding varieties were introduced, which has been attributed to breeders targeting grain yield without accounting for mineral content (55, 56). Proso millet breeding in the US has been limited compared to wheat, so there is an opportunity to consider mineral concentration in the development of new varieties.

Days to heading was negatively correlated with all minerals besides Fe and Ca, indicating that quicker-developing varieties had higher mineral concentration. However, 2018 study of pearl millet found contradictory results, where Zn, P, Cu, and Mn were positively associated with days to 50% flowering (37). Another study of proso millet found no association between days to 50% flowering and Zn, Cu, or Mn (35). A study of common wheat also found no association between Zn and days to heading (50).

The negative correlation observed between mineral concentration and seed size (TSW and SA) could be attributed to the “dilution effect,” where mineral concentration decreases with grain size due to an increase only in the endosperm of the grain and not the bran or germ which contains most minerals (58). A study of mineral concentration in perennial and annual wheat cultivars showed a negative association with TSW and Ca, Cu, and Zn when both perennial and annual cultivars were analyzed, but found no correlation when just perennial lines were analyzed, suggesting that dilution effect may not apply across grains (58). However, our study provides evidence supporting dilution effect in proso millet.

In many crops, associations have been shown with traits important in emergence (such as seed germination and seedling vigor) and size, density, or weight of seeds (59), however, we did not observe association between these traits. A 2014 study showed wheat with large seed size associated with more promising agronomic performance than wheat with small seed size, which is consistent with our results (60). This same study showed wheat emerged from larger seeds also resulted in taller plants, which is also consistent with our results, and that wheat sown from larger seed resulted in higher yield (60). This final association could be assumed from the results of our study, although sown seeds were not measured or included in our correlation.

## 5 Conclusion

This preliminary study of commercially available proso millet varieties grown in the inland PNW showed distinct difference in mineral concentration, seed morphology, and agronomic phenotypes. Varieties from the researcher-run trial showed significant differences for all traits evaluated. Plateau, the variety with the highest mineral concentration was also among the lowest yielding. Until recently, mineral concentration has been historically overlooked by plant breeders. As there has been limited breeding for proso millet in the inland PNW there is an opportunity to incorporate ionomics into breeding goals and selection targets. Breeders will need to consider the potential for a dilution effect when selecting for high-yielding, high-mineral cultivars. As most minerals were positively correlated with one another, increasing specific minerals through breeding may help to improve the overall mineral richness of a cultivar.

Seed morphology has been shown to be associated with germination physiology, nutrient quality, and yield, and can be easily targeted by breeders as they are less impacted by the environment (52). We observed this association in our study, in a positive correlation of grain yield with seed area and thousand seed weight. However, environment and different treatments did appear to impact seed morphology, as we observed varietal differences in seed area, seed eccentricity, thousand seed weight, and seed color by location. Further investigation is required to explain these results, as producer-run trials had a small sample size and high variability in treatments.

Proso millet core collections have high phenotypic and molecular diversity, which means there is considerable potential for genetic improvement in breeding programs (48, 52). Breeding efforts in the US have been limited and those that have occurred have taken place exclusively in Central Great Plains region of the US. While commercially available varieties were successfully grown out in this region in the 2022 season, further research is required to target breeding goals specific to cropping systems in the inland PNW, as well as end-use qualities desired by processors and consumers in the regional food system.

## Data Availability

The raw data supporting the conclusions of this article will be made available by the authors, without undue reservation.
